# In Situ-Derived Bi_4_Ti_3_O_12_-Bi_2_S_3_ Ferroelectric-Semiconductor Heterojunction as a Multifunctional Separator Coating for Lithium–Sulfur Batteries

**DOI:** 10.3390/nano16161016

**Published:** 2026-08-18

**Authors:** Dehang Ren, Yujiang Sun, Yuzhe Zhang, Xiao Sun, Shijie Xu, Jiakai Wang, Yifan Yan, Xuanting Ding, Yongan Yang

**Affiliations:** 1 Institute of Molecular Plus, Department of Chemistry, School of Chemical Engineering and Technology, Tianjin University, Tianjin 300072, China; dehang@tju.edu.cn (D.R.); sunyujiang5813@tju.edu.cn (Y.S.); zyz139@tju.edu.cn (Y.Z.); 2022239018@tju.edu.cn (X.S.); xsj2021239025@tju.edu.cn (S.X.); 2023239031@tju.edu.cn (J.W.); 1022239011@tju.edu.cn (Y.Y.); ding_xuanting@tju.edu.cn (X.D.); 2 Tianjin Key Laboratory of Advanced Caron and Electrochemical Energy Storage, Tianjin 300072, China

**Keywords:** lithium–sulfur batteries, ferroelectric-semiconductor heterojunction, in-situ conversion, separator modification, polysulfide adsorption and catalytic conversion

## Abstract

The practical viability of lithium–sulfur batteries (LSBs) is severely hindered by sluggish liquid–solid conversion kinetics and the polysulfide shuttle effect. Herein, we report an in situ-derived ferroelectric-semiconductor Bi_4_Ti_3_O_12_-Bi_2_S_3_ heterojunction as a multifunctional separator coating. The intimate atomic-level coupling at the heterointerface generates a built-in electric field that, synergizing with the spontaneous ferroelectric polarization of Bi_4_Ti_3_O_12_, structurally intensifies polysulfide chemisorption and lowers the activation energy for bi-directional Li_2_S precipitation/dissociation. Furthermore, the localized polar field appears to homogenize lithium-ion flux, which may contribute to improved lithium anode stability. Consequently, cells featuring the modified separator deliver a high initial capacity of 1172 mAh g^−1^ at 0.5 C and demonstrate good cycling stability over 500 cycles with a low capacity decay rate of 0.096% per cycle. This in situ interfacial engineering offers a promising kinetic regulatory strategy for improving the performance of LSBs.

## 1. Introduction

Lithium–sulfur batteries (LSBs) have garnered significant attention as a next-generation energy storage system owing to their exceptional theoretical energy density, cost-effectiveness, and environmental benignity [1,2,3]. Nevertheless, the road to their practical deployment is obstructed by formidable challenges, predominantly the severe capacity fading and inferior rate capability. These issues stem fundamentally from the intrinsic solid–liquid–solid conversion mechanism of sulfur electrochemistry [4,5,6]. The generation and dissolution of soluble lithium polysulfide intermediates (LiPSs) induce a detrimental “shuttle effect,” wherein they migrate across the separator to the anode, causing irreversible parasitic reactions with the lithium metal, loss of active material, and rapid capacity decay [7]. Concurrently, the sluggish kinetics of the multistep polysulfide conversion and the solid-state Li_2_S nucleation/dissolution processes hinder the full realization of the theoretical capacity, particularly under high current densities.

To surmount these hurdles, integrating electrocatalysts within the LSB cathode or separator architecture has emerged as a viable strategy [8,9,10,11]. An ideal catalyst must possess a dual affinity—a robust anchoring capability for LiPSs to suppress their shuttling, and a high electrocatalytic activity to accelerate their redox transformation. However, these functionalities are often mutually exclusive in single-component materials [12,13]. In this context, heterojunction catalysts, which intimately integrate two materials with disparate physicochemical properties, offer a promising solution by synergizing strong adsorption and high catalytic activity at their well-defined interfaces [14]. Earlier studies have elegantly demonstrated the efficacy of this approach in enhancing LSB cycle life and rate performance. For instance, Zhou et al. synthesized a TiO_2_-TiN heterostructure anchored on graphene (TiO_2_-TiN@G) as an effective separator coating, achieving remarkable long-term durability (73% capacity retention after 2000 cycles) [15]. In a parallel effort, Jing et al. integrated a CoNi@TiO_2_/C heterojunction into LSB separators, endowing the cells with a specific capacity of 856 mAh g^−1^ after 300 cycles at 0.5 C and a low decay rate of merely 0.09% per cycle [16].

Ferroelectric materials, characterized by their switchable spontaneous polarization, constitute a promising class of catalysts for LSBs [17]. Their intrinsic polar surfaces serve as strong electrostatic anchors for polar LiPSs, while the long-range, orientationally tunable polarization field can profoundly influence ion transport and interfacial charge transfer kinetics. These unique attributes have motivated the deployment of ferroelectrics as cathode hosts [18,19,20], separator coatings [21,22,23], and interlayers [24], where the polar surfaces synergize with chemical adsorption mechanisms to suppress polysulfide dissolution and migration, and the reorientable polarization vector reshapes the electric field distribution to facilitate Li^+^ transport and promote uniform lithium deposition.

Despite this promise, early applications of single-phase ferroelectrics such as BaTiO_3_ were constrained by the intrinsically low electronic conductivity of most ferroelectric oxides, which curtails electron supply and retards conversion kinetics [21,25,26,27]. Integrating a ferroelectric phase into a heterojunction architecture overcomes this limitation by coupling the ferroelectric polarization field with the built-in electric field at the heterointerface [28]. This synergy can deliver more efficient polysulfide trapping and catalytic conversion than either single-phase ferroelectrics or conventional heterojunction catalysts. As demonstrated by Xu et al., a TiO_2_/BaTiO_3_@C heterostructure employed as a sulfur host exploited the ferroelectric polarization of BaTiO_3_ for enhanced LiPS adsorption, while the built-in field at the TiO_2_/BaTiO_3_ interface cooperatively accelerated charge transport and lithium-ion diffusion [29]. Critically, such heterostructures can dynamically respond to the evolving electric field during charge/discharge cycles, with the spontaneous polarization orientation and interfacial internal field adjusting in concert to enable real-time regulation of LiPS adsorption and electrocatalysis.

A subsequent breakthrough involved coupling ferroelectrics directly with metallic conductors to address the conductivity constraint at the system level. Ma et al. developed a BaTiO_3_@Ti_3_C_2_ Mott–Schottky heterostructure that integrates the ferroelectricity of BaTiO_3_ with the metallic conductivity of Ti_3_C_2_ MXene for separator modification, delivering stable cycling over 700 cycles with a capacity decay rate of merely 0.056% per cycle [30]. However, such composites are typically synthesized via ex situ physical mixing, which often yields loose, non-ideal interfaces with high contact resistance, thereby compromising the efficiency of the built-in electric field and the full exploitation of the ferroelectric effect. Collectively, while the former oxide–oxide heterojunctions offer intimate interfaces but suffer from poor conductivity, and the latter ferroelectric–metal configurations provide excellent conductivity but lack tight interfacial coupling. A key challenge thus evolves: how to construct a ferroelectric heterojunction with an intimately coupled, seamless interface to achieve optimal synergistic coupling between polarization and catalysis?

Bi_2_S_3_, a p-block metal sulfide semiconductor with good electronic conductivity and excellent electrocatalytic activity, has been widely adopted in LSBs [31,32,33,34,35]. Several Bi_2_S_3_-based heterostructures have been explored for Li–S batteries. For instance, Al-Tahan et al. designed an In_2_S_3_/Bi_2_S_3_@rGO heterostructure as a modified separator/dual-layer sulfur cathode, achieving a high areal capacity of 7.1 mAh cm^−2^ after 200 cycles under a high sulfur loading of 11.53 mg cm^−2^ [31]. Li et al. constructed a Bi_2_S_3_-MoS_2_ heterostructure interlayer, which delivered a rate capability of 841.6 mAh g^−1^ at 5 C and retained 70.3% capacity after 500 cycles at 3 C [33]. Hua et al. demonstrated that among p-block metal sulfide catalysts, Bi_2_S_3_ exhibits outstanding activity for the sulfur reduction reaction, enabling stable cycling at 5 C with 85% capacity retention after 500 cycles [35]. Bi_4_Ti_3_O_12_, a reported ferroelectric oxide [20], has shown promising applications in LSB cathodes: For instance, Zhou et al. demonstrated that Bi_4_Ti_3_O_12_ exhibits spontaneous polarization and can induce an internal electric field beneficial for polysulfide conversion in Li–S batteries. The prepared flower-like Bi_4_Ti_3_O_12_/CNT composite delivered 818 mA h g^−1^ with 83% retention after 100 cycles at 0.5 C [20]. To the best of our knowledge, no Bi_4_Ti_3_O_12_-based heterojunction has been reported for Li–S batteries. Given that single-phase ferroelectric oxides suffer from poor electronic conductivity while single-phase sulfides lack ferroelectric polarization, constructing a heterojunction that couples a ferroelectric with a semiconducting sulfide is expected to synergistically combine their advantages and overcome individual drawbacks. Critically, both materials belong to the bismuth compound family and the former can be directly converted into the latter via a sulfidation reaction, enabling the in situ construction of a tightly coupled heterointerface via a sulfidation reaction—a feature not readily achievable with other ferroelectric–semiconductor combinations [36].

Therefore, inspired by the chemical convertibility between Bi_4_Ti_3_O_12_ and Bi_2_S_3_, we herein propose an in situ conversion strategy to fabricate a tightly integrated Bi_4_Ti_3_O_12_-Bi_2_S_3_ ferroelectric-semiconductor heterojunction as a high-performance separator modifier for LSBs. Partial sulfidation of the Bi_4_Ti_3_O_12_ precursor directly generates the highly conductive, electrocatalytically active Bi_2_S_3_ phase, forging a seamless heterointerface that drives directional electron transfer from Bi_4_Ti_3_O_12_ to Bi_2_S_3_. The resulting heterojunction integrates three functionalities: (i) Bi_2_S_3_ accelerates liquid-to-solid conversion kinetics by reducing the thermodynamic barrier; (ii) the ferroelectric polarization of Bi_4_Ti_3_O_12_ may facilitate Li^+^ transport and promote more uniform lithium deposition by generating a mesoscopic electric field; and (iii) the in situ derived heterointerface and hierarchical porous architecture expose abundant active sites for effective LiPS capture. Consequently, the heterojunction couples the strong polar adsorption of the ferroelectric with the high electrocatalytic activity of the narrow-bandgap semiconductor, enabling efficient polysulfide trapping and accelerated sulfur redox kinetics, while the ferroelectricity of Bi_4_Ti_3_O_12_ simultaneously stabilizes the lithium metal anode. Comprehensive experimental and electrochemical analyses confirm that this design substantially boosts the overall electrochemical performance of LSBs, delivering enhanced stability and rate capability.

## 2. Experimental Section

### 2.1. Chemicals and Materials

All chemicals were used as received without further purification, including bismuth nitrate pentahydrate (Bi(NO_3_)_3_·5H_2_O, Meryer Chemical, Shanghai, China, 99.9%), tetrabutyl titanate (TBOT, Aladdin, Shanghai, China, 98%), sodium hydroxide (NaOH, Tianjin Jiangtian Chemical, Tianjin, China, 98%), sodium hydrosulfide (NaHS, TCL Shanghai, Shanghai, China, 95%), sodium sulfide (Na_2_S, Tianjin Damao Chemical, Tianjin, China, 98%), sublimed sulfur (S, Tianjin Damao Chemical, 99.7%), Ketjen black (KB, Aladdin, 99.9%), poly(vinylidene fluoride) (PVDF, Shanghai D&B, Shanghai, China, 99.9%), N-methyl-2-pyrrolidone (NMP, Energy Chemical, Shanghai, China, 99.9%), lithium sulfide (Li_2_S, Aladdin, 99%), lithium bis(trifluoromethanesulfonyl)imide (LiTFSI, Canrd, Shenzhen, Guangdong, China, 99.9%), tetraethylene glycol dimethyl ether (TEGDME, Aladdin, 99.5%), 1,2-dimethoxyethane (DME, Aladdin, 99.5%), 1,3-dioxolane (DOL, Aladdin, 99%).

### 2.2. Synthesis of Bi_4_Ti_3_O_12_ and Bi_2_S_3_

Bi_4_Ti_3_O_12_ was synthesized via a hydrothermal route. In a typical procedure, 60 mL of 5 M NaOH aqueous solution was prepared using deionized (DI) water, and Bi(NO_3_)_3_·5H_2_O and TBOT were added in a molar ratio of 4:3 under magnetic stirring. After continuous stirring for 30 min, the mixture was ultrasonicated for 20 min. The resulting suspension was transferred into a 100 mL autoclave and heated at 180 °C for 12 h. The solid product was collected by centrifugation, washed alternately with DI water and ethanol for three cycles each, and then dried at 70 °C overnight. For comparison, pristine Bi_2_S_3_ was prepared by adding Bi(NO_3_)_3_·5H_2_O and Na_2_S (molar ratio 2:3) into DI water, followed by hydrothermal treatment at 180 °C for 12 h.

### 2.3. Synthesis of Bi_4_Ti_3_O_12_–Bi_2_S_3_ Heterojunction

The Bi_4_Ti_3_O_12_–Bi_2_S_3_ heterojunction was prepared by an in situ sulfidation method. In brief, 0.2 mmol of Bi_4_Ti_3_O_12_ powder was dispersed in 30 mL of DI water and transferred into a 100 mL autoclave. A designated amount of NaHS was dissolved in water and added dropwise to bring the total volume to 60 mL. The autoclave was sealed and heated at 180 °C for 12 h. The resulting black product was collected by centrifugation, washed alternately with DI water and ethanol, and dried at 70 °C overnight. By adjusting the amount of NaHS, Bi_4_Ti_3_O_12_–Bi_2_S_3_ composites with different phase ratios were obtained.

### 2.4. Preparation of Modified Separators

A commercial polypropylene separator (Celgard 2325, Celgard, Charlotte, NC, USA) was modified by a doctor-blade coating method. Bi_4_Ti_3_O_12_–Bi_2_S_3_, Ketjen black (KB), and PVDF were mixed at a weight ratio of 7:2:1 in an agate mortar, and then NMP was added dropwise under magnetic stirring to form a homogeneous slurry. The slurry was coated onto the separator surface using a doctor blade and dried at 60 °C overnight; the coated separator was punched into disks of 16 mm diameter. The mass loading of the coating on the separator, determined by weighing the separator before and after coating, was approximately 0.18 mg cm^−2^. For control experiments, separators modified solely with Bi_4_Ti_3_O_12_ or Bi_2_S_3_ were fabricated by the same procedure, each replacing the heterojunction with an equal mass of the respective material.

### 2.5. Li_2_S Nucleation Tests

A 0.2 M Li_2_S_8_ catholyte was prepared by dissolving Li_2_S and sublimed sulfur (molar ratio 1:7) in 1.0 M LiTFSI/TEGDME electrolyte under magnetic stirring at 60 °C for 48 h. Using carbon paper as the cathode current collector and lithium foil as the anode, 20 µL of the 0.2 M Li_2_S_8_ catholyte was dropped onto the cathode side, while 20 µL of Li_2_S_8_-free 1.0 M LiTFSI/TEGDME was placed on the anode side. CR2032 coin cells (Canrd, Shenzhen, Guangdong, China) were assembled with either a commercial or a modified separator. After sufficient standing to ensure complete electrolyte wetting, the cells were first discharged at a constant current of 0.112 mA to 2.10 V, and then subjected to potentiostatic discharge at 2.05 V until the current fell below 0.01 mA.

### 2.6. Li_2_S Dissolution Tests

Li_2_S dissolution tests were performed on the same cells following the nucleation experiment. The cells were discharged at a constant current of 0.112 mA to 1.80 V to convert the polysulfides into solid lithium sulfide, and then charged at a constant voltage of 2.40 V until the current dropped below 0.01 mA for the complete dissolution of Li_2_S.

### 2.7. Synthesis of Li_2_S_6_ Solution and Visualized Adsorption Test

Li_2_S and sublimed sulfur (molar ratio 1:5) were added to a mixed solvent of DME and DOL (1:1, *v*/*v*) and stirred at 60 °C for 48 h to obtain a 5 mM Li_2_S_6_ solution. For the adsorption test, 20 mg of each catalyst was separately immersed in 2 mL of the above Li_2_S_6_ solution. After standing for a certain period, the supernatant was characterized by UV–Vis spectrophotometry.

### 2.8. Li_2_S_6_ Symmetric Cell Assembly and Tests

A 5 mM Li_2_S_6_ electrolyte was prepared by dissolving Li_2_S and sublimed sulfur (molar ratio 1:5) in a mixed solvent of DME and DOL (1:1, *v*/*v*) containing 1.0 M LiTFSI. Symmetric cells were assembled using two identical carbon paper electrodes, each wetted with 20 µL of the Li_2_S_6_ electrolyte, and a commercial or modified separator was sandwiched between them. After resting for adequate electrolyte infiltration, CV and EIS measurements were performed to evaluate the electrocatalytic activity.

### 2.9. Preparation of S/KB Cathode

The S/KB composite was prepared by a melt-diffusion strategy. Sublimed sulfur and Ketjen black (mass ratio 75:25) were intimately ground in an agate mortar, transferred into a sealed glass vial, and heated at 155 °C for 12 h. The resulting S/KB active material was mixed with additional KB and PVDF at a mass ratio of 80:10:10 in NMP to form a uniform slurry. The slurry was coated onto a carbon-coated aluminum foil current collector, vacuum-dried at 60 °C overnight, and punched into disks of 12 mm diameter. The sulfur loading was controlled within 0.8–1.1 mg cm^−2^.

### 2.10. Cell Assembly and Electrochemical Tests

CR2032 coin cells were assembled with the as-prepared S/KB cathode (12 mm diameter, sulfur loading 0.8–1.1 mg cm^−2^), lithium foil (~450 µm thickness, 16 mm diameter, N/P ratio > 50) as the anode and a commercial (Celgard 2325) or modified separator. The electrolyte was 1.0 M LiTFSI in a mixed solvent of DOL and DME (1:1, *v*/*v*) containing 2 wt% LiNO_3_ as additive; the electrolyte volume was fixed at 30 µL and the corresponding E/S ratio was 24–33 µL mg^−1^. Li/Li symmetric cells were constructed by sandwiching a separator between two lithium foils using the same electrolyte. All cells were assembled in an argon-filled glovebox with H_2_O and O_2_ levels below 0.1 ppm. Galvanostatic charge–discharge tests were performed on a LAND CT2001A battery test system within a voltage window of 1.7–2.8 V at various C-rates (1 C = 1675 mAh g^−1^). Cyclic voltammetry (CV) and electrochemical impedance spectroscopy (EIS) were carried out on a CHI660E electrochemical workstation. All electrochemical measurements were conducted at room temperature (25 °C), and the specific capacities were calculated based on the mass of sulfur.

### 2.11. Materials Characterization

Morphology and elemental analysis were performed using a Apreo S LoVac field-emission scanning electron microscope (FEI Company, Brno, Czech Republic) equipped with an energy-dispersive X-ray spectroscopy (EDS) detector. Transmission electron microscopy (TEM) characterization was conducted on a Talos F200X field-emission microscope (Thermo Fisher Scientific, Hillsboro, OR, USA) fitted with a Super-X G2 EDS detector (Thermo Fisher Scientific). X-ray diffraction (XRD) patterns were recorded on a Rigaku SmartLab 9 kW diffractometer (Akishima-shi, Tokyo, Japan) using Cu K_α_ radiation (λ = 0.15406 nm) at a scanning rate of 20° min^−1^, with an operating voltage of 45 kV and a current of 200 mA. X-ray photoelectron spectroscopy (XPS) was performed on an ESCALAB 250Xi spectrometer (Thermo Fisher Scientific); binding energies were calibrated by referencing the C 1s peak to 284.8 eV. The specific surface area and pore size distribution were measured on an ASAP 2460 analyzer (Micromeritics, Norcross, GA, USA). Samples were degassed at 200 °C under vacuum for 6 h prior to measurement, and N_2_ adsorption–desorption isotherms were recorded at liquid-nitrogen temperature. The specific surface area was calculated by the Brunauer–Emmett–Teller (BET) method, and the pore size distribution was derived from the Barrett–Joyner–Halenda (BJH) model. UV–Vis absorption spectra were collected on a Evolution 201 spectrophotometer (Thermo Fisher Scientific), with the background signal of the DME/DOL solvent subtracted.

## 3. Results and Discussion

**Figure 1:**
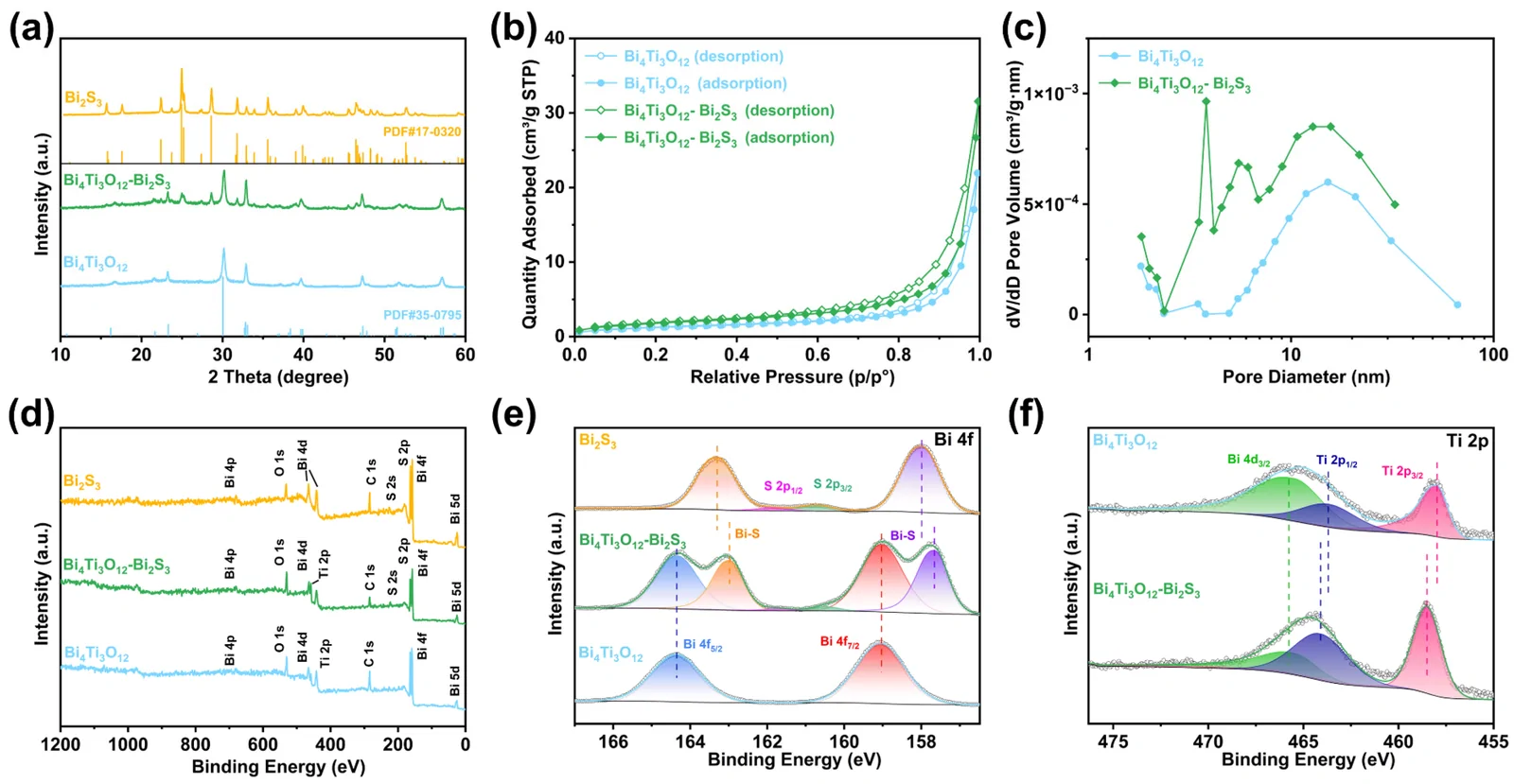
Basic characterization of Bi_4_Ti_3_O_12_–Bi_2_S_3_ and the two reference samples: (**a**) XRD patterns of Bi_4_Ti_3_O_12_–Bi_2_S_3_, Bi_4_Ti_3_O_12_, and Bi_2_S_3_. (**b**) N_2_ adsorption–desorption isotherms of Bi_4_Ti_3_O_12_ and Bi_4_Ti_3_O_12_–Bi_2_S_3_. (**c**) Pore size distributions of Bi_4_Ti_3_O_12_ and Bi_4_Ti_3_O_12_–Bi_2_S_3_. (**d**) XPS survey spectra of Bi_4_Ti_3_O_12_–Bi_2_S_3_, Bi_4_Ti_3_O_12_, and Bi_2_S_3_. (**e**) High-resolution Bi 4f spectra. (**f**) High-resolution Ti 2p spectra.

The Bi_4_Ti_3_O_12_–Bi_2_S_3_ heterostructure was synthesized by in situ sulfidation of Bi_4_Ti_3_O_12_. For comparison, pristine Bi_4_Ti_3_O_12_ and Bi_2_S_3_ were prepared via hydrothermal reactions. Detailed procedures are given in the Section 2 and Figure S1. X-ray diffraction (XRD) was employed to unravel the crystal structures of the three products. As shown in Figure 1a, the pattern of Bi_4_Ti_3_O_12_–Bi_2_S_3_ contains diffraction peaks from both Bi_4_Ti_3_O_12_ (PDF#35-0795) and Bi_2_S_3_ (PDF#17-0320) [20,31]. This confirms that part of the Bi_4_Ti_3_O_12_ precursor was converted into Bi_2_S_3_ by the sulfidation reaction, yielding a biphasic heterostructure. The diffraction patterns of the single-phase references align perfectly with their respective standards, verifying their high phase purity.

To elucidate the evolution of the porous architecture post-sulfidation, N_2_ adsorption–desorption analyses were conducted. The isotherms of the Bi_4_Ti_3_O_12_ precursor and Bi_4_Ti_3_O_12_–Bi_2_S_3_ are shown in Figure 1b. Both curves display type IV isotherms, characteristic of mesoporous structures. The BET specific surface areas are 4.61 m^2^ g^−1^ for Bi_4_Ti_3_O_12_ and 6.80 m^2^ g^−1^ for Bi_4_Ti_3_O_12_–Bi_2_S_3_. Concurrently, the pore size distribution (Figure 1c) reveals that while Bi_4_Ti_3_O_12_ predominantly features pores in the 10–30 nm regime, the Bi_4_Ti_3_O_12_–Bi_2_S_3_ composite develops two additional fine mesopore populations centered at ~3 and ~5 nm. This enriched hierarchical porosity is beneficial for facilitating electrolyte penetration and exposing abundant active heterointerfaces for polysulfide chemisorption and subsequent catalytic conversion.

X-ray photoelectron spectroscopy (XPS) was carried out to analyze the surface composition and chemical states. The survey spectrum (Figure 1d) confirms the presence of Bi, Ti, O, S, and C in Bi_4_Ti_3_O_12_–Bi_2_S_3_. In the high-resolution Bi 4f spectrum (Figure 1e), two doublets are resolved. The peaks at 159.03 and 164.35 eV are assigned to Bi 4f_7/2_ and Bi 4f_5/2_ of Bi_4_Ti_3_O_12_ [36], while the peaks at 157.66 and 162.98 eV originate from Bi–S bonds in Bi_2_S_3_ [37]. Compared with pristine Bi_2_S_3_, the Bi–S peaks in the heterostructure shift noticeably to lower binding energy; the Bi 4f peaks of Bi_4_Ti_3_O_12_ remain essentially unchanged. In the Ti 2p spectrum (Figure 1f), the Ti 2p_3/2_ and Ti 2p_1/2_ peaks appear at 458.50 and 464.10 eV, respectively, consistent with Ti^4+^. An additional peak at 465.76 eV is ascribed to Bi 4d_3/2_ [20,38]. Relative to single-phase Bi_4_Ti_3_O_12_, the Ti 2p binding energy in the heterostructure shifts to higher values. These complementary shifts indicate significant interfacial electron transfer from Bi_4_Ti_3_O_12_ to Bi_2_S_3_ across the in situ formed heterointerface, which is a direct signature of establishing a built-in electric field, as reported in numerous studies that have attributed such XPS peak shifts as compelling evidence for built-in electric fields in heterojunction catalysts [39,40,41]. Such a built-in field not only accelerates electron transfer during electrocatalysis, but also redistributes interfacial charge—creating electron-deficient and electron-rich regions that generate additional adsorption/active sites—thereby imparting catalytic performance superior to that of the single-component materials [39,40]. While the XPS peak shifts provide evidence for the built-in electric field, direct visualization of the internal electric field (e.g., via Kelvin probe force microscopy (KPFM) or piezoresponse force microscopy (PFM)) would further consolidate this conclusion and could be conducted in the future if such tools were accessible.

**Figure 2:**
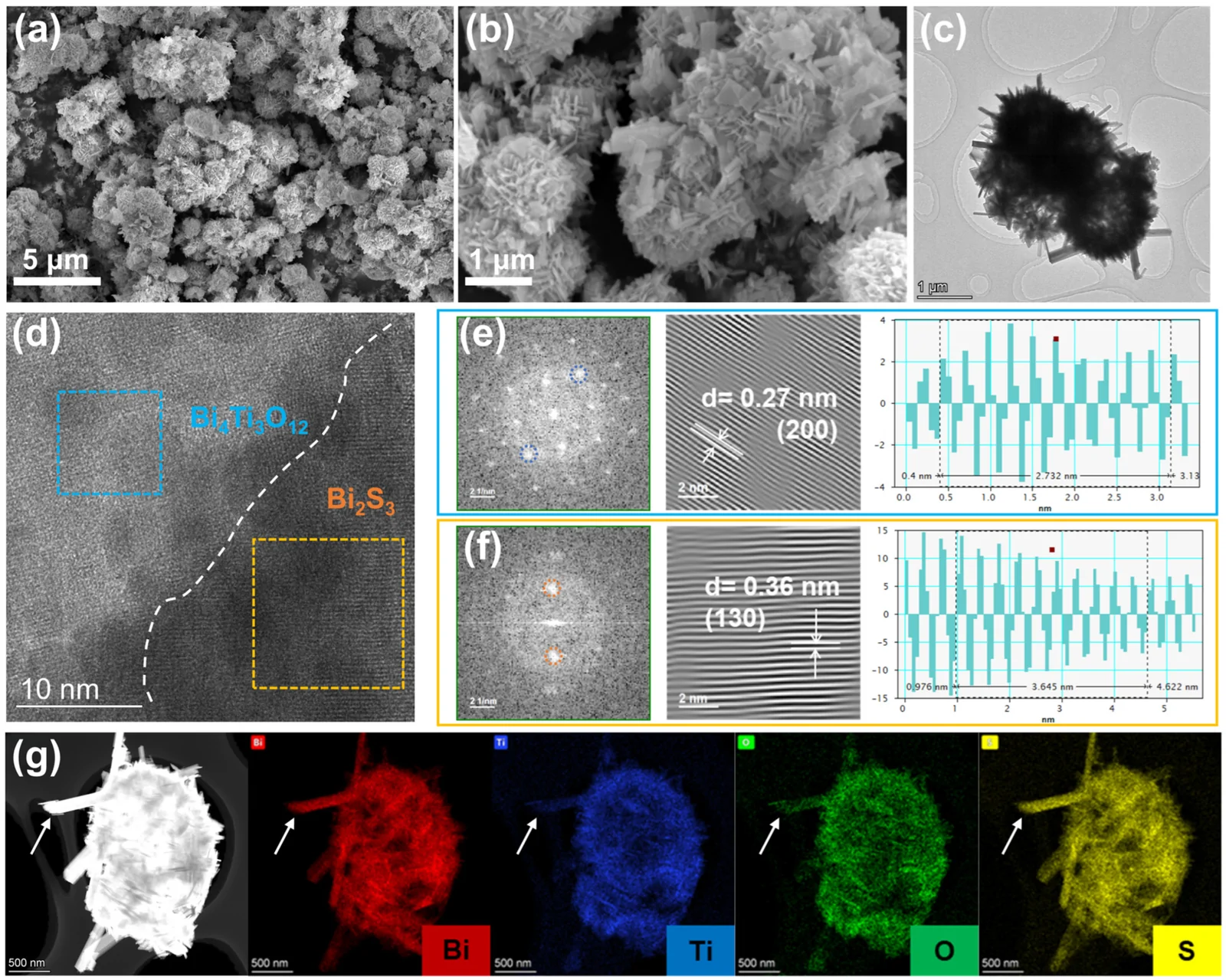
Electron microscopy characterization of the as-synthesized Bi_4_Ti_3_O_12_–Bi_2_S_3_: (**a**,**b**) SEM images at magnifications of 16,000× and 60,000×, respectively. (**c**) Low-magnification TEM image. (**d**) High-resolution TEM image. (**e**,**f**) FFT pattern, IFFT lattice image, and lattice distance profile of selected areas corresponding to Bi_4_Ti_3_O_12_ and Bi_2_S_3_, respectively. (**g**) EDS elemental maps of Bi, O, Ti, and S for Bi_4_Ti_3_O_12_–Bi_2_S_3_.

The morphology was examined by scanning electron microscopy (SEM). The SEM images (Figure 2a,b) show that Bi_4_Ti_3_O_12_–Bi_2_S_3_ consists of flower-like spheres assembled from nanosheets, with nanorods grown on the surface. This architecture is visually corroborated by transmission electron microscopy (TEM, Figure 2c), which agrees well with the SEM observations. High-resolution TEM (HRTEM) was employed to resolve the internal microstructure (Figure 2d). Fast Fourier transform (FFT) and inverse FFT (IFFT) analyses of the HRTEM image unravel distinct lattice fringes with spacings of 0.27 nm and 0.36 nm, corresponding to the (200) plane of Bi_4_Ti_3_O_12_ and the (130) plane of Bi_2_S_3_ [31,42], respectively (Figure 2e,f). The coexistence of these lattice planes from both phases aligns with the XRD results, confirming the successful formation of the biphasic composite. Moreover, the HRTEM image reveals a strongly coupled interface between the two phases (highlighted by the white line in Figure 2d), demonstrating that a chemically bonded heterointerface was created through the in situ sulfidation reaction, consistent with the XPS findings [41]. Furthermore, energy-dispersive X-ray spectroscopy (EDS) elemental mapping (Figure 2g) delineates the spatial configuration of the heterostructure. While the core region exhibits a homogeneous dispersion of Bi, Ti, O, and S—indicating the presence of abundant and well-distributed Bi_4_Ti_3_O_12_/Bi_2_S_3_ interfaces—the protruding nanorods (highlighted by white arrows) present concentrated Bi and S signals with a conspicuous absence of Ti and O. This elemental segregation confirms that the surface-grown nanorods are predominantly pure Bi_2_S_3_ phase [43]. Such a rationally designed hierarchical structure not only secures massive built-in electric fields at the core but also exposes highly conductive Bi_2_S_3_ nanorods on the surface, ensuring maximal accessibility for polysulfide entrapment and catalytic conversion.

To evaluate the catalytic efficacy of the different materials in lithium–sulfur batteries, each catalyst was uniformly blended with Ketjen black and PVDF to form a slurry. The obtained homogeneous slurry was subsequently coated onto commercial polypropylene (PP) separators to construct functional interfacial layers. Cross-sectional SEM and EDS mapping (Figure S2) confirm a highly uniform elemental distribution within an ultrathin coating layer of approximately 3 µm. Such a thin, functional layer facilitates electrolyte infiltration and rapid ion transport, thereby introducing negligible parasitic weight and preserving the overall energy density of the cell. For systematic mechanistic analysis, the separators modified with Bi_4_Ti_3_O_12_–Bi_2_S_3_, Bi_4_Ti_3_O_12_, and Bi_2_S_3_, together with the bare PP separator, are denoted as BTO-BS, BTO, BS, and PP, respectively.

**Figure 3:**
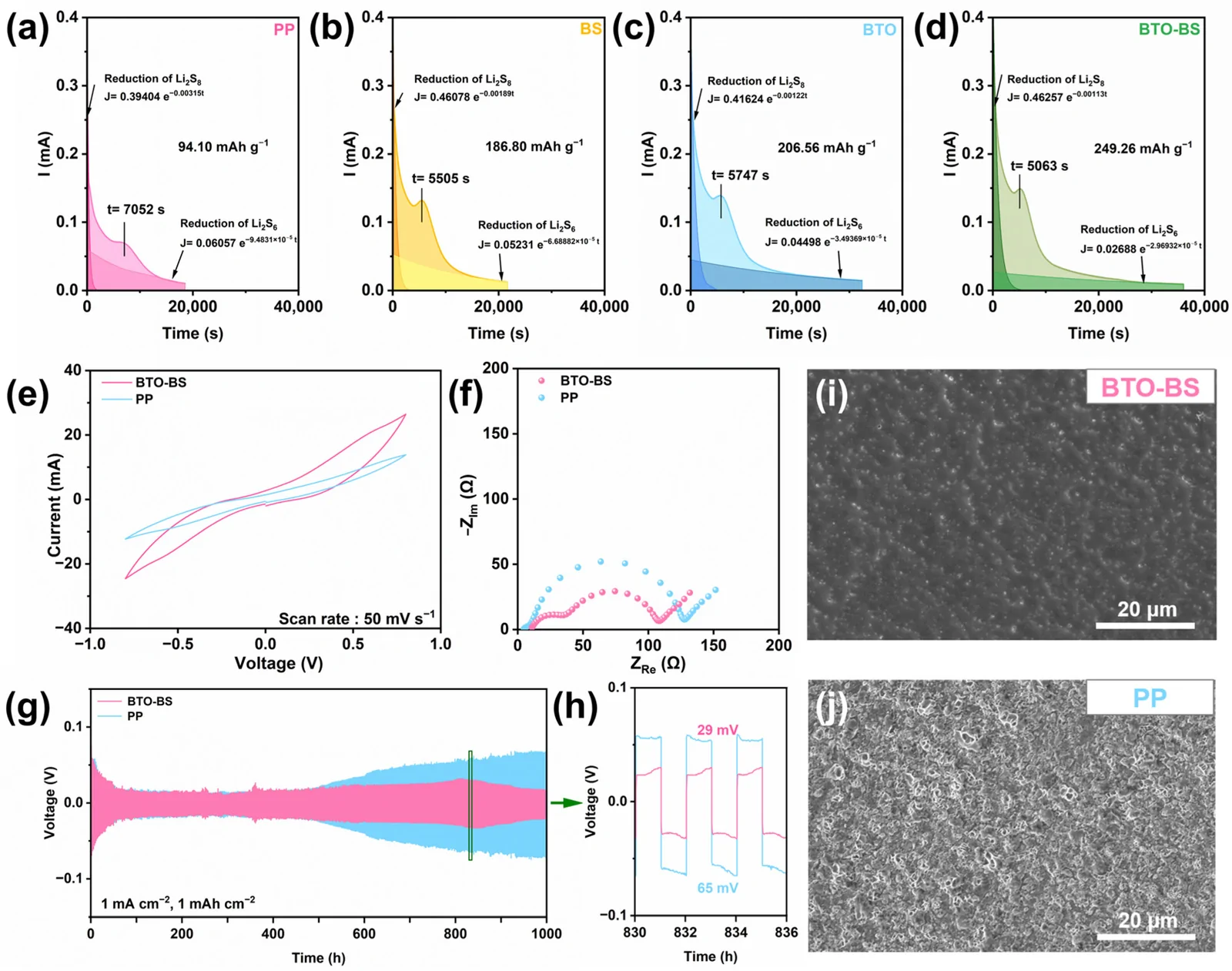
Electrocatalytic and electrochemical characterization of the modified separators. (**a**–**d**) Potentiostatic discharge I–t curves of the Li_2_S nucleation tests. (**e**) Cyclic voltammetry curves of symmetric cells at 50 mV s^−1^. (**f**) EIS results. (**g**,**h**) Voltage–time profiles of Li/Li symmetric cells assembled with PP and modified separators. (**i**,**j**) SEM images of the Li anode in Li/Li symmetric cells with BTO-BS and PP separators after cycling, respectively.

The liquid-to-solid phase transition of polysulfides, a kinetically sluggish bottleneck in sulfur electrochemistry, was rigorously probed via potentiostatic Li_2_S nucleation and dissolution tests using a precisely controlled Li_2_S_8_ catholyte [44]. During the nucleation process (discharged galvanostatically to 2.10 V and held at 2.05 V), the resulting I–t transients (Figure 3a–d) reveal that BTO-BS achieves the earliest nucleation current peak at 5063 s. This induction time is shorter than those of both single-component catalysts and the pristine PP, suggesting a reduced activation energy barrier for Li_2_S precipitation. Quantitative integration of the potentiostatic curves further reveals that BTO-BS delivers the highest Li_2_S deposition capacity of 249.26 mAh g^−1^, outperforming BTO (206.56 mAh g^−1^), BS (186.80 mAh g^−1^), and PP (94.10 mAh g^−1^). Notably, the better performance of pristine BTO over BS indicates that the ferroelectric polarization of BTO helps enrich local polysulfide concentration, which thermodynamically favors phase conversion.

For the reverse kinetic evaluation (Li_2_S dissolution), cells were galvanostatically discharged to 1.80 V and subsequently polarized at 2.40 V. As elucidated in Figure S3, BTO-BS exhibits a higher dissolution peak current of 2.06 mA within merely 198 s, considerably outperforming BTO (1.74 mA, 246 s), BS (1.31 mA, 302 s), and PP (0.69 mA, 681 s). Furthermore, BTO-BS records the highest reversible Li_2_S dissolution capacity of 371.27 mAh g^−1^. Collectively, these kinetic assays indicate that the BTO-BS heterostructure functions as an effective bidirectional electrocatalyst, accelerating the complete liquid–solid interconversion [13]. Specifically, the built-in electric field drives directional electron transfer from Bi_4_Ti_3_O_12_ to Bi_2_S_3_, facilitating rapid electron supply to active sites. Meanwhile, the ferroelectric polarization generates a local electric field that attracts polar polysulfide anions, enriching their local concentration at the heterointerface, and also promotes Li^+^ transport, accelerating Li_2_S nucleation and dissolution.

To identify the optimal heterojunction composition, the feeding molar ratio of Bi_4_Ti_3_O_12_ to the sulfidation agent (NaHS) was varied at 1:1, 1:3, and 1:5 (denoted as BTO-BS-1:*x*). The XRD patterns, presented in Figure S4, validate that the diffraction peaks of Bi_2_S_3_ intensify with increasing NaHS content, reflecting a progressively larger Bi_2_S_3_ fraction. Morphological comparison (Figure S5) reveals that under high sulfur-source conditions, the Bi_2_S_3_ nanorods grow longer and tend to segregate from the Bi_4_Ti_3_O_12_ matrix. Combining with the SEM images of single-phase Bi_2_S_3_ (Figure S6) and Bi_4_Ti_3_O_12_ (Figure S7), the structural transformation can be inferred as follows: mild sulfidation largely preserves the intrinsic flower-like spherical framework of BTO while inducing the in situ growth of Bi_2_S_3_ nanorods on the exposed surfaces. However, an excessive sulfur precursor triggers the overgrowth and severe segregation of Bi_2_S_3_ nanorods from the BTO matrix, which severely disrupts the intimate interfacial contact. Conversely, suboptimal sulfidation fails to construct a percolating interfacial network, whereas excessive sulfidation leads to phase segregation and poor interfacial contact. Potentiostatic Li_2_S dissolution evaluation (Figure S8) confirms that the cell with the BTO-BS-1:3 separator reaches the highest peak current (2.06 mA) in the shortest response time (198 s), manifesting optimized catalytic kinetics. Accordingly, the BTO-BS-1:3 sample was selected for all subsequent electrochemical tests and mechanistic analyses.

The rapid sequestration of soluble polysulfides is essential to suppress the shuttle effect and mitigate active-material loss from the cathode. The intrinsic chemisorption affinities of the various catalysts toward polysulfides were visually and quantitatively assessed using a static Li_2_S_6_ adsorption assay performed in an argon-filled glovebox. Equal masses of BTO-BS, BTO, and BS were each added to identical volumes of the Li_2_S_6_ solution and allowed to stand for 12 h. The supernatants were then analyzed by UV–Vis spectroscopy. As shown in Figure S9a, the absorption peak intensity—proportional to the residual Li_2_S_6_ concentration—decreases in the order BS > BTO > BTO-BS, and all three are lower than that of the blank control without any catalyst. The corresponding optical photographs (Figure S9b) visually corroborate this quantitative analysis; the solution treated with BTO-BS undergoes obvious decolorization, becoming almost visually transparent, signifying highly effective polysulfide entrapment. While BTO also induces visible fading, the single-phase BS exhibits the weakest color change. This superiority can be attributed to a synergistic dual-advantage: first, the built-in electric field enhances chemical binding; second, the protruding Bi_2_S_3_ nanorods increase the specific surface area, providing more anchoring sites. These adsorption/anchoring effects are primarily responsible for suppressing the polysulfide shuttle.

To explicitly decouple and evaluate the intrinsic electrocatalytic activity toward liquid-phase polysulfide conversion, Li_2_S_6_ symmetric cells engineered with identical carbon-paper working electrodes were systematically investigated. Cyclic voltammetry (CV) was performed at a scan rate of 50 mV s^−1^. As shown in Figure 3e, the symmetric cell with BTO-BS delivers a higher current response than the other cells, suggesting faster polysulfide conversion kinetics and more efficient charge transfer, which is conducive to releasing higher capacity. This enhanced current response can be partly attributed to the hierarchical porous structure of the BTO-BS heterojunction (Figure 1c). The interconnected bimodal pores centered at ~3 nm and ~5 nm serve multiple functions, such as increasing the specific surface area, ensuring rapid electrolyte infiltration, providing short Li^+^ diffusion pathways, and exposing abundant active sites. Consequently, this hierarchical architecture improves electrolyte wettability, reduces concentration polarization, and facilitates polysulfide conversion, thereby delivering a higher current density. This kinetic enhancement is further substantiated by electrochemical impedance spectroscopy (EIS) and distribution of relaxation times (DRT) analysis. Typically, the semicircle in the mid-frequency region of EIS corresponds to the charge transfer resistance [45]. At open-circuit voltage (Figure 3f), the BTO-BS exhibits the smaller charge transfer resistance (108 Ω), while the PP shows a value of 127 Ω. The DRT profiles (Figure S10) resolve five distinct relaxation processes. Following the assignment method of Li et al. for symmetric cells [46], τ_1_, τ_2_, and τ_3_ are associated with contact resistances, τ_4_ reflects the charge-transfer resistance of the liquid–liquid electrochemical reaction, and τ_5_ corresponds to polysulfide diffusion. The peak value of τ_4_ is 39.7 Ω for BTO-BS, significantly smaller than the 88.9 Ω for the PP separator. The DRT analysis thus confirms that BTO-BS reduces the charge-transfer resistance and accelerates the liquid-phase polysulfide redox interconversion. This marked kinetic enhancement is primarily attributed to the synergistic effect of the heterointerface, where a built-in electric field not only strengthens the chemical affinity for polar LiPSs but also accelerates charge transfer during their catalytic conversion.

Beyond sulfur electrochemistry, the interfacial stability of the lithium metal anode was scrutinized via prolonged galvanostatic cycling of Li/Li symmetric cells, evaluated at a current density of 1 mA cm^−2^ with a fixed areal capacity of 1 mAh cm^−2^. As shown in Figure 3g, the cell with the bare polypropylene separator exhibits a pronounced voltage increase after only 500 h, whereas the cell with the modified separator sustains stable cycling for 1000 h. After 800 h, the symmetric cell with the modified separator shows an overpotential of about 29 mV, less than half that of the bare separator (65 mV, Figure 3h), demonstrating improved stability toward lithium metal. To directly visualize the beneficial effect of the separator on lithium deposition, the surface morphology of lithium anode after cycling was examined by SEM. As shown in Figure 3i, the lithium anode paired with the BTO-BS modified separator displays a continuous, smooth, and dense surface without observable dendrites or cracks. In stark contrast, the lithium surface in contact with the bare PP separator (Figure 3j) is rough and uneven, covered with mossy deposits and irregular needle-like Li dendrites. This chaotic morphology indicates uncontrolled nucleation and growth, which stems from the non-uniform Li^+^ flux through the pristine polyolefin separator. The compact and uniform Li deposition observed with the BTO-BS separator is consistent with a more homogenized Li^+^ flux, which likely contributes to reduced dendrite formation. This improvement can be rationalized by the ferroelectric nature of Bi_4_Ti_3_O_12_ in the BTO-BS heterostructure. Under the operating internal electric field, the spontaneous polarization of BTO dynamically aligns to generate a localized, homogenizing polar field [24]. This integrated field may help regulate the Li^+^ flux and reduce the formation of localized high-current-density hotspots, thereby mitigating dendritic lithium growth and supporting more stable long-term cycling. It should be noted that while the improved Li deposition morphology and symmetric-cell stability are evident, the underlying mechanism—particularly the role of ferroelectric polarization in regulating Li^+^ flux to improve the cycling stability of the Li-metal anode—remains partially speculative. Direct measurements such as operando impedance evolution or Li/Cu Coulombic efficiency could be employed in the future to fully elucidate the underlying mechanism.

**Figure 4:**
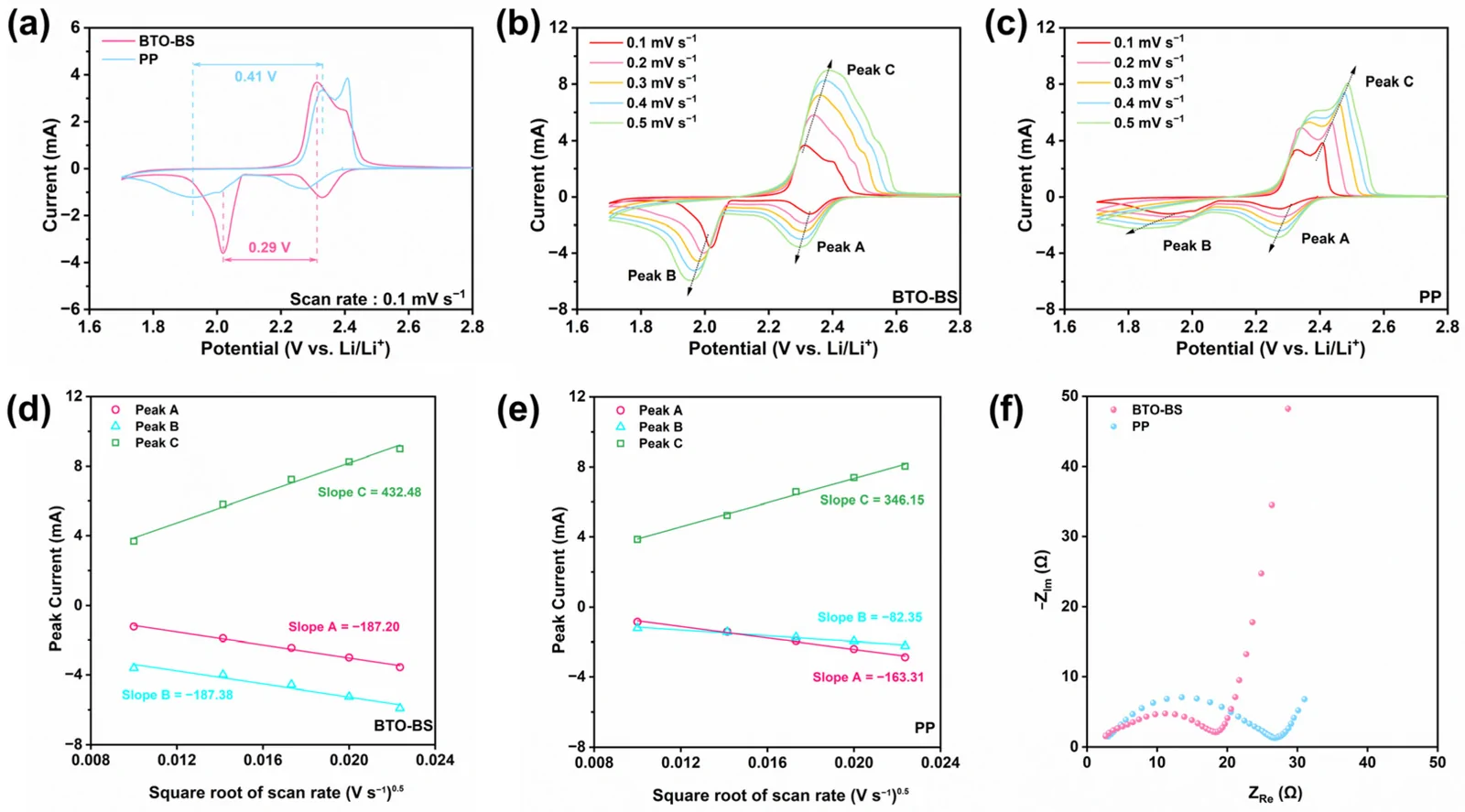
Electrochemical performance of Li–S full cells: (**a**) CV curves at a scan rate of 0.1 mV s^−1^. CV curves at various scan rates for (**b**) BTO-BS and (**c**) PP. (**d**,**e**) Linear relationship between peak current and square root of scan rate. (**f**) EIS spectra.

The impact of the rationally designed catalyst on full-cell performance was assessed using Li–S coin cells. Figure 4a compares the cyclic voltammetry profiles at a scan rate of 0.1 mV s^−1^ within the voltage range of 1.7–2.8 V. Notably, the cell incorporating BTO-BS delivers sharper and more prominent redox peaks. Compared to the bare PP separator, the oxidation peak of BTO-BS is negatively shifted from 2.33 V to 2.31 V, while the second reduction peak is positively shifted from 1.92 V to 2.02 V. Consequently, the voltage gap (∆E) between the reduction and oxidation peaks is only 0.29 V for BTO-BS, smaller than the 0.41 V observed for PP. These features indicate that the heterostructure effectively lowers the thermodynamic energy barriers, suppresses electrochemical polarization, and enhances the bidirectional polysulfide redox kinetics.

To further elucidate the mass-transfer kinetics, CV curves were further recorded at varied scan rates from 0.1 to 0.5 mV s^−1^ (Figure 4b,c). Following the classical Randles-Sevcik equation, the peak currents are plotted against the square root of the scan rate. As delineated by the linear fits in Figure 4d,e, the BTO-BS cell exhibits steeper slopes than the control cell, suggesting a substantially accelerated lithium-ion diffusion coefficient (D_Li+_) within the catalytic matrix [47]. It should be noted that the classical Randles–Sevcik equation is strictly valid for simple diffusion-controlled processes, whereas Li–S electrochemistry involves complex multistep conversion reactions and phase transformations. Therefore, the diffusion coefficients calculated herein should be regarded as apparent values that reflect the combined effect of multiple steps. Although the equation may not fully capture the intricate kinetics, the relative comparison of the calculated values between different separator systems still provides a meaningful qualitative indication of the overall Li^+^ transport capability and reaction kinetics. The interfacial charge-transfer dynamics were further probed via electrochemical impedance spectroscopy (EIS) coupled with distribution of relaxation times (DRT) analysis (Figure 4f). For the full-cell DRT analysis (Figure S11), the assignment follows Soni et al. [48]. The DRT spectra effectively deconvolve the complex impedance into specific electrochemical processes: τ_1_ represents electric double-layer relaxation, τ_2_ corresponds to the solid electrolyte interphase (SEI), τ_3_ denotes charge-transfer resistance, and τ_4_ alongside τ_5_ are ascribed to mass diffusion. Crucially, the characteristic τ_3_ peak value for the BTO-BS cell is 1.79 Ω, lower than that of the PP counterpart (1.93 Ω). Collectively, these systematic kinetic evaluations indicate that the Li-S battery engineered with the BTO-BS heterostructure benefits from highly expedited redox kinetics and superior interfacial charge-transfer capabilities, which directly contribute to the rate capability.

**Figure 5:**
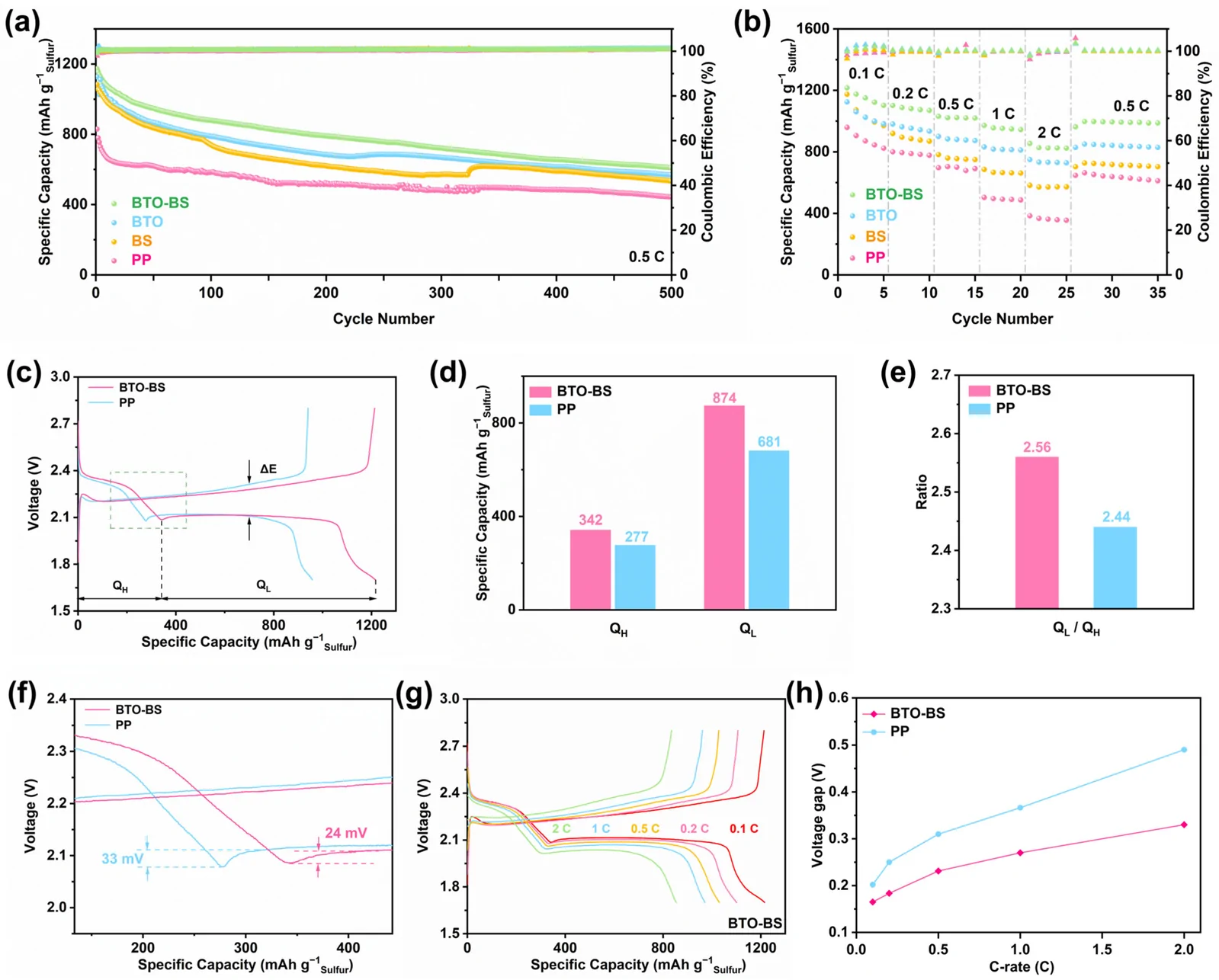
Long-term cycling, rate performance, and charge–discharge analysis of Li–S batteries: (**a**) Cycling performance at 0.5 C for Li–S cells with different separators. (**b**) Rate capability from 0.1 to 2 C. (**c**) Charge/discharge curves at 0.1 C. (**d**) Q_H_ and Q_L_ values for BTO-BS and PP cells derived from the 0.1 C discharge curves. (**e**) Q_L_/Q_H_ ratios for the corresponding cells. (**f**) Enlarged view around the Li_2_S nucleation point. (**g**) Galvanostatic discharge–charge voltage profiles of BTO-BS at different rates. (**h**) Voltage gap as a function of C-rate.

To validate the practical viability and catalytic efficacy of the heterostructure, the long-term cycling stability and rate capability of Li–S coin cells were evaluated. Figure 5a displays the galvanostatic charge–discharge (GCD) performance at 0.5 C. The BTO-BS cell delivers a high initial discharge capacity of 1172 mAh g^−1^ and a high reversible capacity of 609 mAh g^−1^ after 500 cycles, outperforming BTO (568 mAh g^−1^), BS (533 mAh g^−1^), and PP (443 mAh g^−1^). Correspondingly, the capacity decay rate is only 0.096% per cycle, highlighting the improved cycling stability enabled by the heterostructure. One might be concerned whether trapping lithium polysulfides by the separator coating under lean-electrolyte conditions would have a negative effect on the cycling process or not. Indeed, the noticeable capacity decay observed over 500 cycles in Figure 5a suggests that such negative effects may occur under prolonged operation. Possible factors include continuous electrolyte consumption due to parasitic side reactions and irreversible loss of active polysulfides. These issues are known to become more pronounced under low electrolyte/sulfur (E/S) ratios. Since the present tests were conducted with relatively high E/S ratios (24–33 µL mg^−1^) and moderate sulfur loading (0.8–1.1 mg cm^−2^), a systematic investigation of the coating’s effect under practical lean-electrolyte and high-loading conditions is left for future work, additional modification strategies, such as electrolyte additives or anode protection layers, may also be needed to synergistically suppress capacity decay under such demanding conditions. The rate performance evaluated at current densities from 0.1 to 2 C is shown in Figure 5b. BTO-BS delivers a specific capacity of 853.9 mAh g^−1^ even at 2 C, demonstrating good high-rate capability. Upon reverting the current density to 0.5 C, the capacity promptly rebounds to 997 mAh g^−1^, confirming the outstanding structural integrity of the catalyst and the electrochemical reversibility of the sulfur species. A quantitative comparison of the key electrochemical performance metrics for Li–S batteries with BTO-BS, BTO, BS, and PP separators is summarized in Table S1.

The fundamental origins of this enhanced performance can be elucidated by dissecting the GCD profiles. The galvanostatic charge–discharge profiles recorded at 0.1 C (Figure 5c) exhibit the characteristic two-stage discharge plateaus (~2.33 V and ~2.12 V) and a single charge plateau (~2.26 V) of Li–S chemistry. The voltage hysteresis between the charge and discharge plateaus is only 0.165 V for BTO-BS, smaller than the 0.202 V of the PP separator (Figure 5c), reflecting reduced polarization and accelerated polysulfide conversion kinetics.

To quantify the catalytic impact on specific reaction stages, the capacities contributed by the high-voltage plateau (Q_H_, reduction in solid sulfur to soluble polysulfides) and the low-voltage plateau (Q_L_, kinetically sluggish liquid-to-solid conversion) were systematically decoupled (Figure 5d,e). The BTO-BS cell delivers a Q_H_ of 342 mAh g^−1^ (an increase of 65 mAh g^−1^ over PP), indicating highly efficient initial sulfur activation. Consequently, it unlocks a massive Q_L_ of 874 mAh g^−1^, pushing the Q_L_/Q_H_ ratio to 2.56—closer to the theoretical limit of 3.0 compared to the PP cell (2.44). This suggests that the heterojunction profoundly drives the liquid-to-solid phase transition approaching completion [49]. This catalytic propulsion is fundamentally corroborated by the Li_2_S nucleation barrier, which can be identified in the discharge profile as the distinct voltage dip between the first and second plateaus (Figure 5f). The nucleation barrier manifests that the BTO-BS interface effectively reduces the nucleation overpotential to 24 mV (versus 33 mV for PP), indicating that the heterointerface provides abundant lithiophilic/sulfiophilic sites to thermodynamically favor and accelerate 3D Li_2_S deposition.

Moreover, the good kinetic resilience of the BTO-BS heterostructure is manifested under high-flux operating conditions. As depicted in the multi-rate GCD curves (Figure 5g), the BTO-BS cell maintains the quintessential two-plateau discharge behavior with only marginal polarization increments, even at an aggressive rate of 2 C. In stark contrast, the PP cell exhibits severely deteriorated kinetics at 2 C (Figure S12), characterized by high overpotentials and significant distortion of the voltage plateaus. Figure 5h plots the voltage gap between the charge and discharge plateaus as a function of C-rate. It is evident that the BTO-BS cell not only operates at lower absolute overpotentials but also features a reduced polarization slope, again demonstrating its ability to sustain fast redox kinetics under high-rate operation [50]. The superior characteristics can be deconvoluted into two contributions: (i) enhanced adsorption/anchoring of polysulfides by the polar Bi_4_Ti_3_O_12_ surface and the built-in electric field, which suppresses the shuttle effect and minimizes active material loss; and (ii) accelerated catalytic conversion of polysulfides, particularly the rate-determining Li_2_S nucleation/dissolution steps, which improves sulfur utilization and reduces polarization. Both effects are enabled by the BTO-BS heterojunction. The results establish BTO-BS as a promising catalytic material for Li–S batteries.

## 4. Conclusions

In summary, an in situ sulfidation strategy is developed to construct a Bi_4_Ti_3_O_12_–Bi_2_S_3_ ferroelectric-semiconductor heterojunction as a highly efficient kinetic modulator for LSBs. The intimately coupled interface generates a built-in electric field, working synergistically with the spontaneous polarization of Bi_4_Ti_3_O_12_, contributing to strong polysulfide anchoring and bidirectional catalysis of the critical Li_2_S nucleation/dissolution steps. The ferroelectric polarization may also help homogenize Li^+^ flux, which appears beneficial for the stability of the lithium metal anode. Consequently, the optimized BTO-BS heterostructure endows Li–S cells with good rate capability—delivering 853.9 mAh g^−1^ even at 2 C—and good long-term cycling stability, retaining 609 mAh g^−1^ after 500 cycles at 0.5 C with a capacity decay rate of only 0.096% per cycle. This work focuses on a mechanistic analysis by conducting the electrochemical evaluations under moderate sulfur loading and electrolyte-rich conditions, paving the way for validating practical applications in future work. Table S2 provides a direct performance comparison of the BTO-BS modified separator with recently reported ferroelectric-based, Bi-based, and other heterostructure materials in Li–S batteries [51,52]. The comparison confirms that our BTO-BS heterojunction exhibits competitive cycling stability and rate capability. This work demonstrates a rational strategy for coupling ferroelectric fields with heterojunction engineering to address kinetic bottlenecks in advanced energy storage systems.

## Data Availability

The original contributions presented in this study are included in the article/Supplementary Material. Further inquiries can be directed to the corresponding author.

## Supplementary Materials

The following supporting information can be downloaded at: https://www.mdpi.com/article/10.3390/nano16161016/s1, Figure S1: Schematic illustration of synthesizing Bi_4_Ti_3_O_12_-Bi_2_S_3_ through an in-situ sulfidation process; Figure S2: SEM and EDS characterizations of the Bi_4_Ti_3_O_12_-Bi_2_S_3_ modified separator; Figure S3: Potentiostatic charge profiles of Li_2_S dissolution tests of four types of separators; Figure S4: XRD patterns of Bi_4_Ti_3_O_12_-Bi_2_S_3_ obtained with different sodium hydrosulfide dosages of 1:1 (blue), 1:3 (green) and1:5 (orange); Figure S5: SEM images of Bi_4_Ti_3_O_12_-Bi_2_S_3_ obtained with different sodium hydrosulfide dosages of 1:1 (a), 1:3 (b) and 1:5 (c); Figure S6: Four representative SEM images of the single component Bi_2_S_3_; Figure S7: Four representative SEM images of the single component Bi_4_Ti_3_O_12_; Figure S8: Potentiostatic charge profiles of Li_2_S dissolution test of Bi_4_Ti_3_O_12_-Bi_2_S_3_ obtained with different sodium hydrosulfide dosages of 1:1 (blue), 1:3 (green) and1:5 (orange); Figure S9: (a) UV–Vis absorbance spectra of the Li_2_S_6_ solution after 12 h adsorption with different samples. (b) Optical photos showing the Li_2_S_6_-adsorbing capability of BTO, BTO-BS, and BS samples after soaked them in a Li_2_S_6_/DOL-DME solution (blank) for 12 h; Figure S10: DRT transformation of the EIS plots shown in Figure 3g, for Li_2_S_6_ symmetric cells using BTO-BS and PP, respectively; Figure S11: DRT transformation of the EIS plots shown in Figure 4d, for Li-S full cells using BTO-BS and PP, respectively; Figure S12: The galvanostatic charge-discharge voltage profiles of PP at different rates from 0.1 to 2 C; Table S1: Electrochemical performance comparison of BTO-BS, BTO, BS, and PP separators in Li–S batteries; Table S2: Comparison of recently reported ferroelectric, Bi-based, and other heterostructure materials in Li–S batteries.
