# Supplementary material for: In Situ-Derived Bi4Ti3O12-Bi2S3 Ferroelectric-Semiconductor Heterojunction as a Multifunctional Separator Coating for Lithium–Sulfur Batteries
Source: Nanomaterials (Basel). 2026 Aug 18;16(16):1016. doi: 10.3390/nano16161016 (PMC13516686; doi:10.3390/nano16161016)
Supplement: Supplementary file 1 [file nanomaterials-16-01016-s001.zip › nanomaterials-4338090-supplementary.pdf]

Supporting Information

In-Situ Derived $Bi_4Ti_3O_{12}$-Bi_2S_3 Ferroelectric Heterojunction as a Multifunctional Separator Coating for Lithium-Sulfur Batteries

Dehang Ren[#a], Yujiang Sun[#a], Yuzhe Zhang [a], Xiao Sun [a], Shijie Xu [a], Jiakai Wang [a], Yifan Yan [a], Xuanting Ding [a], Yongan Yang[*a,b]

[a]Institute of Molecular Plus, Department of Chemistry, School of Chemical Engineering and Technology, Tianjin University, Tianjin 300072, China.

[b]Tianjin Key Laboratory of Advanced Caron and Electrochemical Energy Storage, Tianjin 300072, China.

[#]These authors contributed equally to this work.

[*]Corresponding author: Y. Yang (revned_yang@tju.edu.cn)

Supporting Information

Supplementary Figures

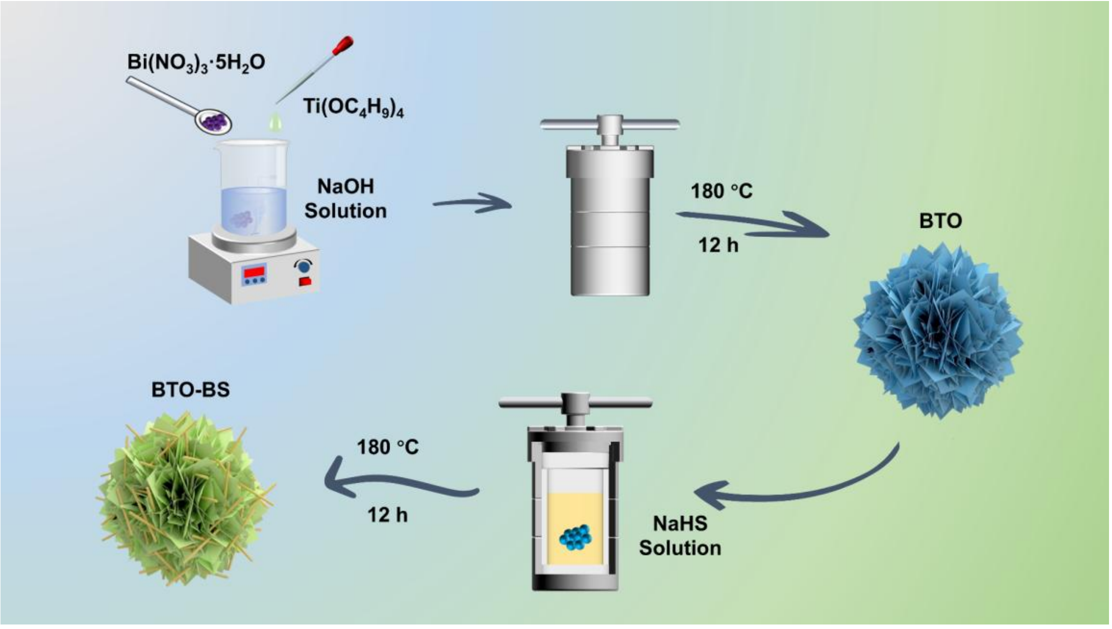

Figure S1. Schematic illustration of synthesizing $Bi_4Ti_3O_{12}$-Bi_2S_3 through an in-situ sulfidation process.

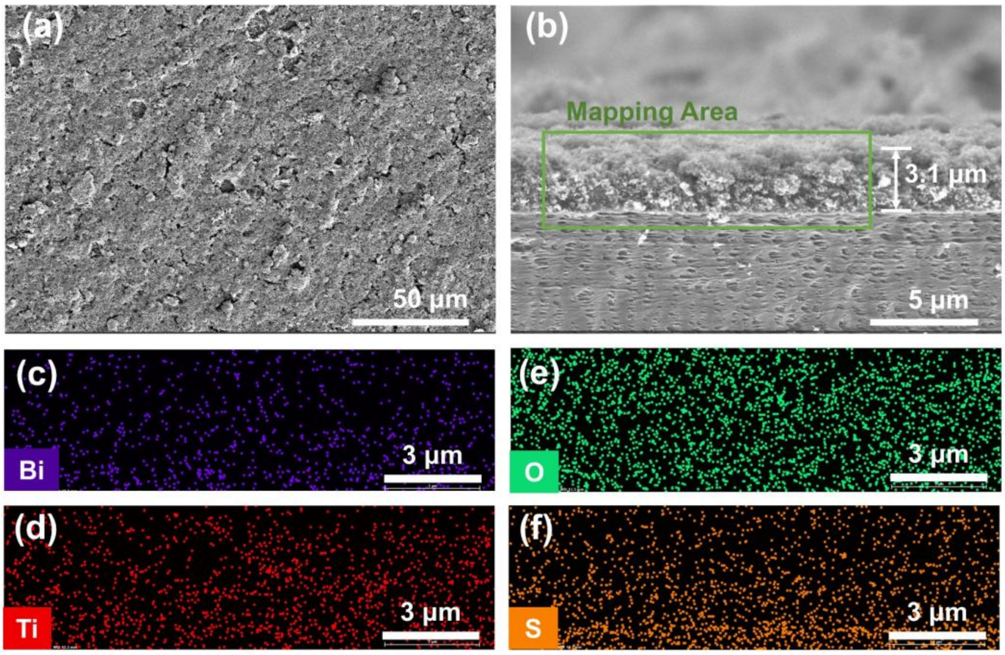

Figure S2. SEM and EDS characterizations of the $Bi_4Ti_3O_{12}$-Bi_2S_3 modified separator. (a) SEM image showing the top surface morphology; (b) SEM image showing the cross-section morphology; (c-f) EDS mapping profiles of elements Bi, Ti, O and S for the marked area in panel b.

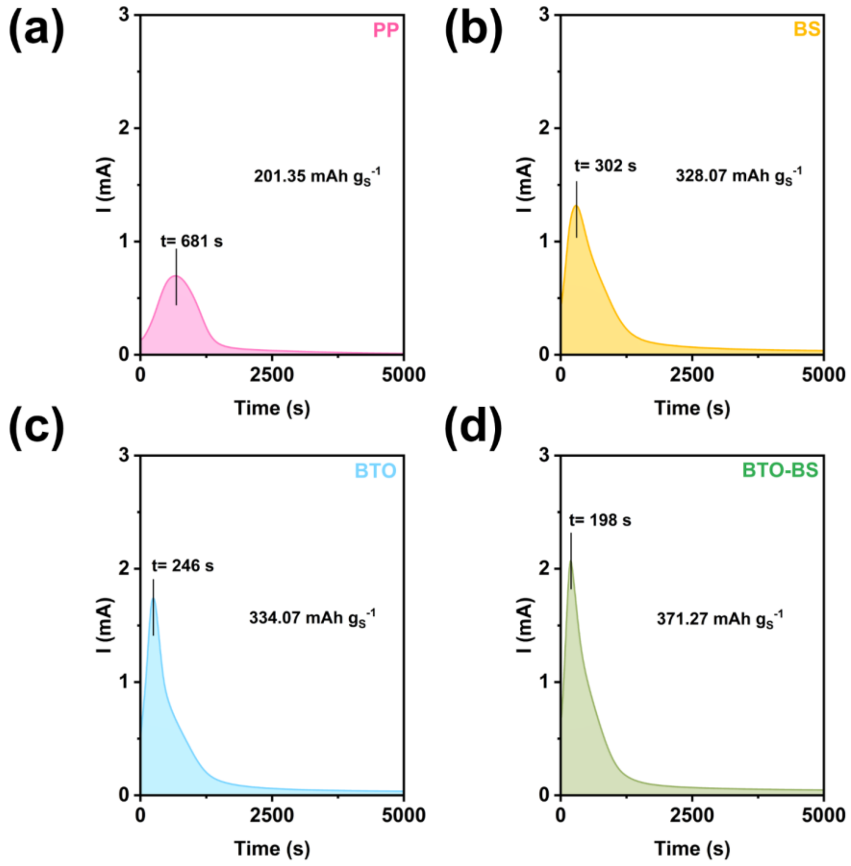

Figure S3. Potentiostatic charge profiles of Li_2S dissolution tests of four types of separators. (a) PP, (b) BS, (c) BTO and (d) BTO-BS.

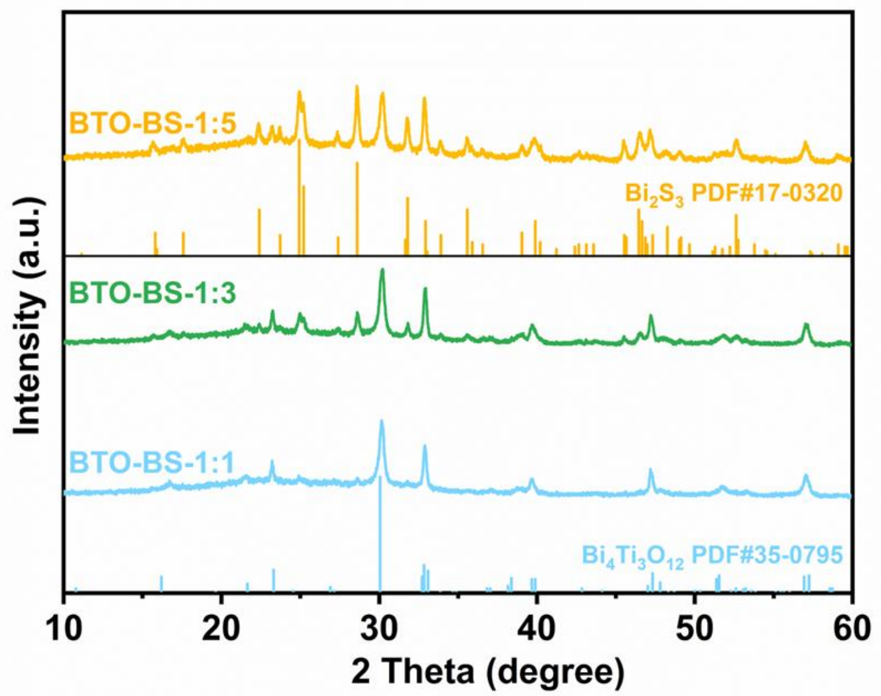

Figure S4. XRD patterns of $Bi_4Ti_3O_{12}$-Bi_2S_3 obtained with different sodium hydrosulfide dosages of 1:1 (blue), 1:3 (green) and1:5 (orange).

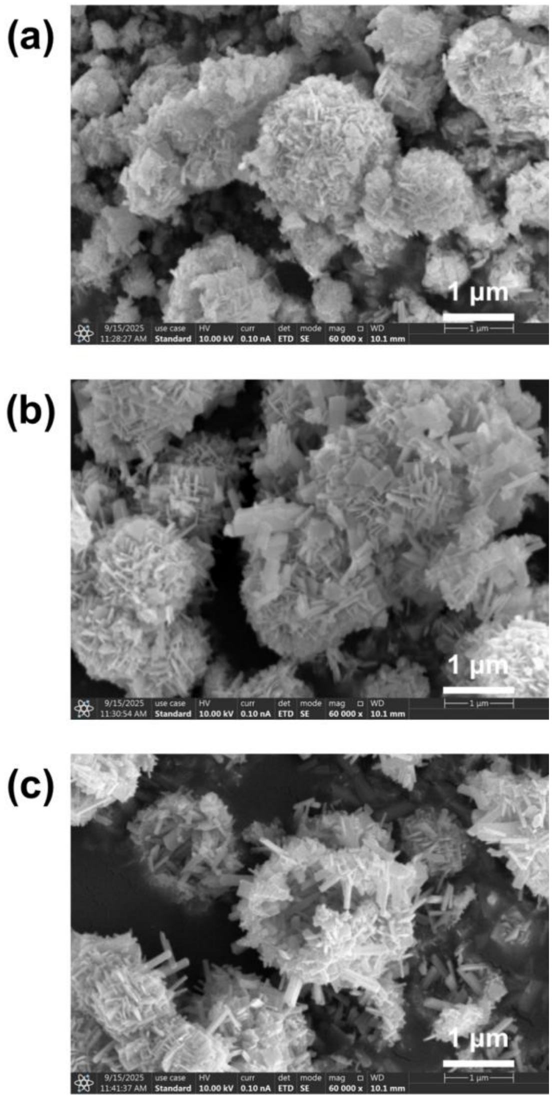

Figure S5. SEM images of $Bi_4Ti_3O_{12}$-Bi_2S_3 obtained with different sodium hydrosulfide dosages of 1:1 (a), 1:3(b) and1:5 (c).

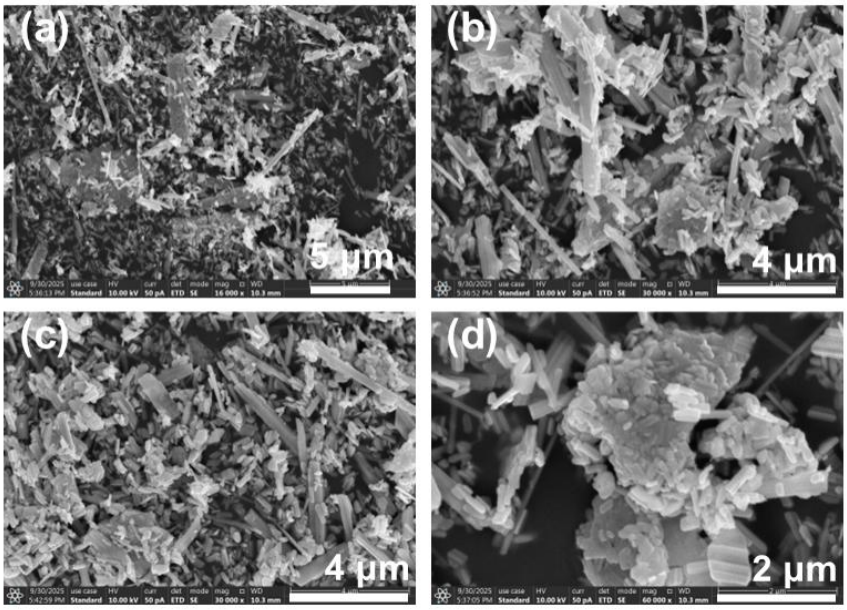

Figure S6. Four representative SEM images of the single component Bi_2S_3.

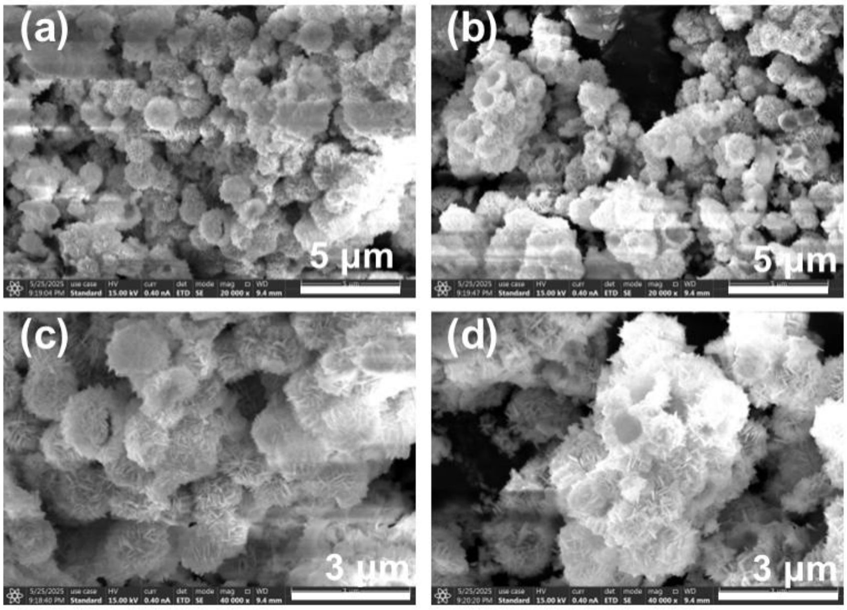

Figure S7. Four representative SEM images of the single component $Bi_4Ti_3O_{12}$.

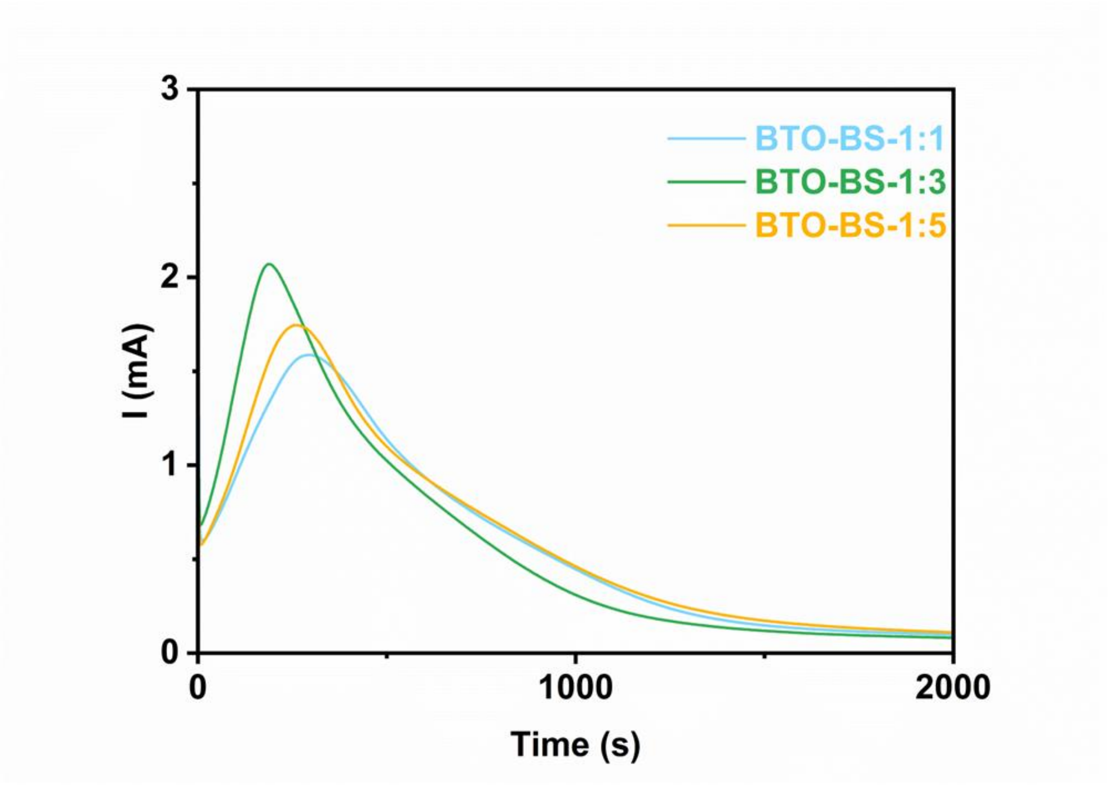

Figure S8. Potentiostatic charge profiles of Li_2S dissolution test of $Bi_4Ti_3O_{12}$-Bi_2S_3 obtained with different sodium hydrosulfide dosages of 1:1 (blue), 1:3 (green) and1:5 (orange).

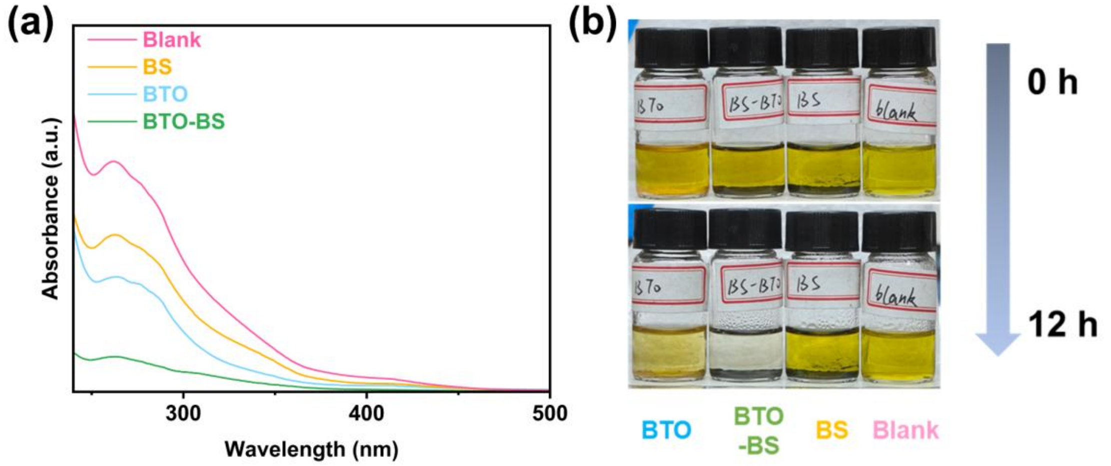

Figure S9. (a) UV–Vis absorbance spectra of the Li_2S_6 solution after 12 h adsorption with different samples. (b) Optical photos showing the Li_2S_6-adsorbing capability of BTO, BTO-BS, and BS samples after soaked them in a Li_2S_6/DOL-DME solution (blank) for 12 h.

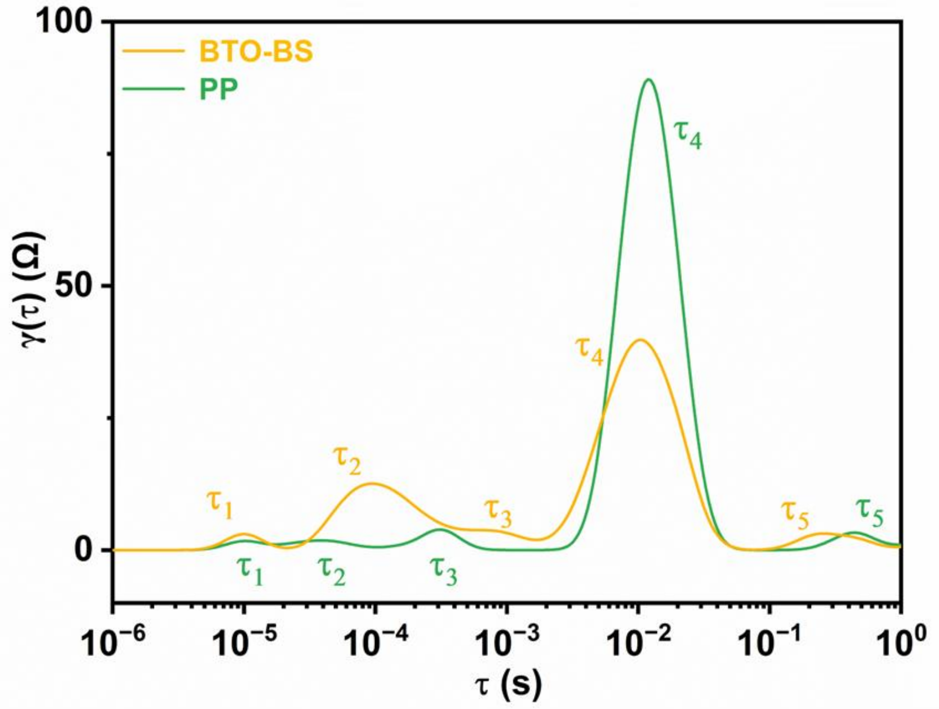

Figure S10. DRT transformation of the EIS plots shown in Fig. 3(g), for Li_2S_6 symmetric cells using BTO-BS and PP, respectively.

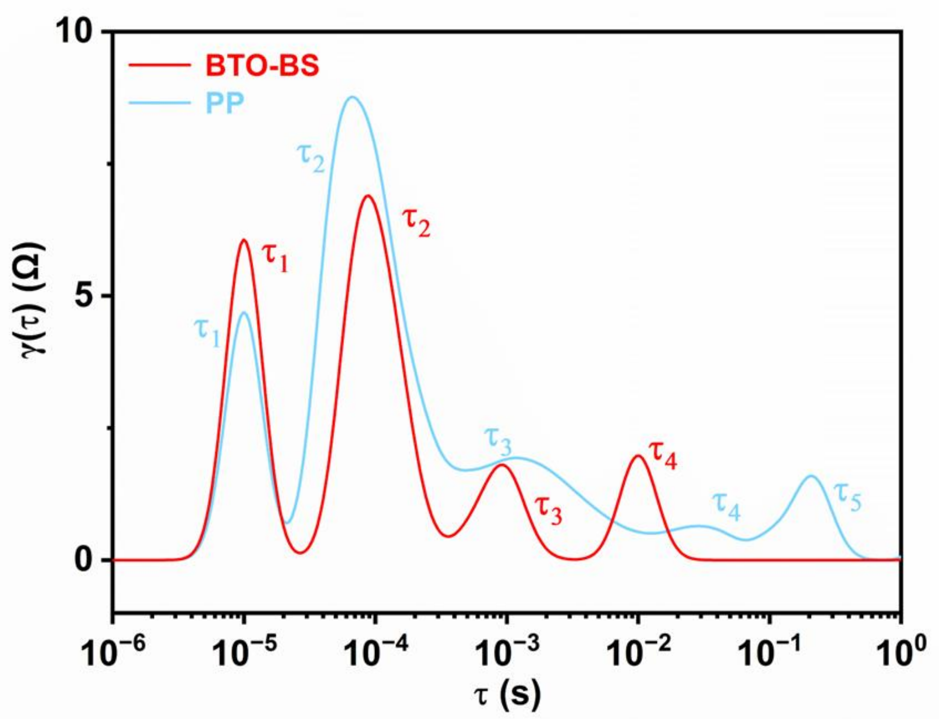

Figure S11. DRT transformation of the EIS plots shown in Fig. 4(d), for Li-S full cells using BTO-BS and PP, respectively.

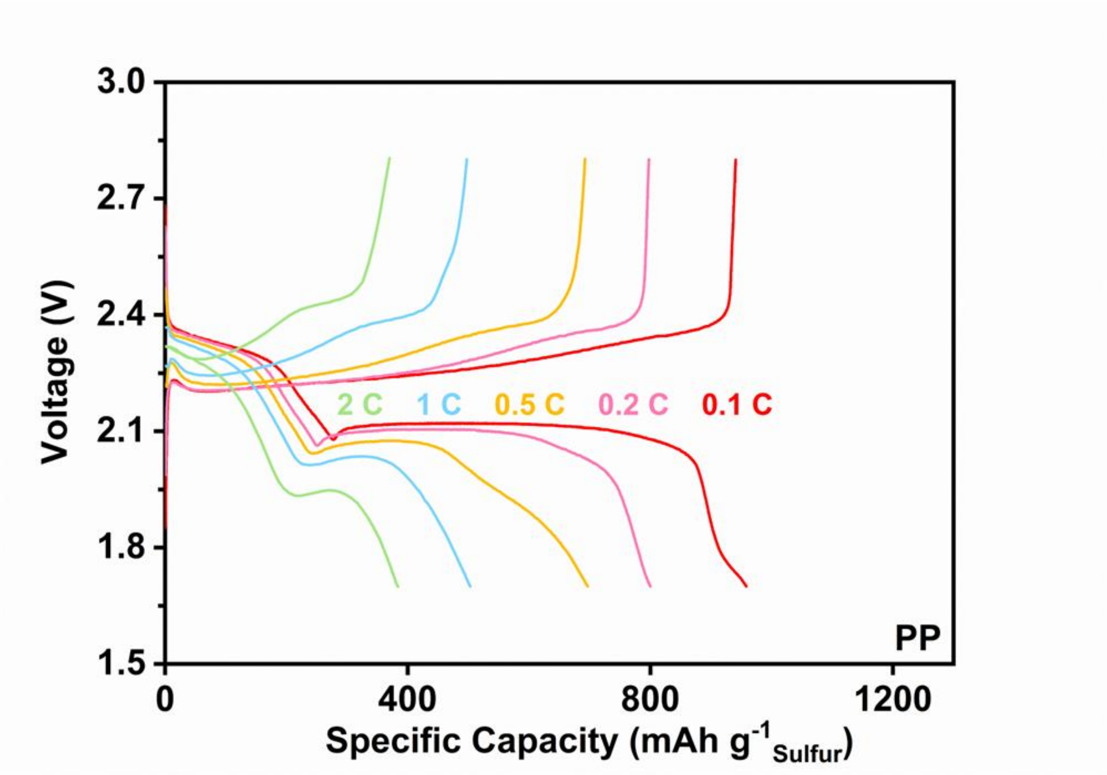

Figure S12. The galvanostatic charge-discharge voltage profiles of PP at different rates from 0.1 to 2 C.

Supporting Information

Table S1. Electrochemical performance comparison of BTO-BS, BTO, BS, and PP separators in Li–S batteries.

Sample	Initial discharge capacity /0.5 C (mAh g^{-1})	Reversible capacity after 500 cycles (mAh g^{-1})	Decay rate per cycle	Discharge capacity at 1 C	Discharge capacity at 2 C
BTO-BS	1172	609	0.096%	971	854
BTO	1127	568	0.099%	831	750
BS	1083	533	0.102%	685	582
PP	829	443	0.093%	503	384

Table S2. Comparison of recently reported ferroelectric, Bi-based, and other heterostructure materials in Li–S batteries.

	Typology	S loading (mg cm^{-2})	C-rate	Discharge capacity (mAh g^{-1}) / Cycle number	Decay rate per cycle	Ref
**$Bi_4Ti_3O_{12}$-Bi_2S_3**	**Separator coating**	**0.8-1.1**	**0.5 C**	**609/500**	**0.096%**	**This work**
$CoNi@TiO_2/C$	Separator coating	1.856	0.5 C	768/150	0.129%	[16]
$Bi_4Ti_3O_{12}/CNT$	Sulfur host	1.0-1.2	0.5 C	818/100	0.16%	[20]
		2.5	1 C	588/100	0.094%	
$BaTiO_3@TiO_x$	Separator coating	1.5	0.5 C	931/100	0.2%	[22]
		2.5	1 C	621/150	0.167%	
$BaTiO_3@PNF$-MoS_2	Interlayer	1.2-1.5	2 C	318/4000	0.015%	[24]
		6.3	1 C	372/400	0.084%	
$TiO_2/BaTiO_3@C$	Sulfur host	~1.0	1 C	534/400	0.075%	[29]
$BaTiO_3@Ti_3C_2$	Separator coating	~1.4	0.5 C	826/100	0.117%	[30]
		2.36	0.2 C	727/140	0.188%	
		4.98	0.1 C	601/140	0.243%	
Bi_2S_3/rGO	Separator coating	~1.5	0.5 C	605/300	0.122%	[34]
VS_2/MoS_2	Electrocatalyst	1.7	0.2 C	721/500	0.08%	[45]
Ni_3N/Nb_4N_5	Separator coating	1.0-1.5	0.2 C	796/200	0.25%	[51]
$BiFeO_3/TiO_2$	Separator coating	2.0	1 C	754/800	0.02%	[52]
